# Expression of Muscle-Specific MiRNA 206 in the Progression of Disease in a Murine SMA Model

**DOI:** 10.1371/journal.pone.0128560

**Published:** 2015-06-01

**Authors:** Valeria Valsecchi, Marina Boido, Elena De Amicis, Antonio Piras, Alessandro Vercelli

**Affiliations:** Neuroscience Institute Cavalieri Ottolenghi, Department of Neuroscience, University of Turin, Turin, Italy; Federico II University of Naples, ITALY

## Abstract

Spinal muscular atrophy (SMA) is a severe neuromuscular disease, the most common in infancy, and the third one among young people under 18 years. The major pathological landmark of SMA is a selective degeneration of lower motor neurons, resulting in progressive skeletal muscle denervation, atrophy, and paralysis. Recently, it has been shown that specific or general changes in the activity of ribonucleoprotein containing micro RNAs (miRNAs) play a role in the development of SMA. Additionally miRNA-206 has been shown to be required for efficient regeneration of neuromuscular synapses after acute nerve injury in an ALS mouse model. Therefore, we correlated the morphology and the architecture of the neuromuscular junctions (NMJs) of quadriceps, a muscle affected in the early stage of the disease, with the expression levels of miRNA-206 in a mouse model of intermediate SMA (SMAII), one of the most frequently used experimental model. Our results showed a decrease in the percentage of type II fibers, an increase in atrophic muscle fibers and a remarkable accumulation of neurofilament (NF) in the pre-synaptic terminal of the NMJs in the quadriceps of SMAII mice. Furthermore, molecular investigation showed a direct link between miRNA-206-HDAC4-FGFBP1, and in particular, a strong up-regulation of this pathway in the late phase of the disease. We propose that miRNA-206 is activated as survival endogenous mechanism, although not sufficient to rescue the integrity of motor neurons. We speculate that early modulation of miRNA-206 expression might delay SMA neurodegenerative pathway and that miRNA-206 could be an innovative, still relatively unexplored, therapeutic target for SMA.

## Introduction

Spinal muscular atrophy (SMA) is a severe neuromuscular disease, the most common in infancy, and the third one among young people under 18 years. The frequency is 8–11 per 100.000 live births [1,2], but one person over 35/50 carries its autosomal recessive gene. Clinical features range from the most severe form (SMAI) to the mildest one (SMAIV), in which patients retain a normal life of relation and have a normal life expectancy. The pathological landmark of SMA is the selective degeneration of lower motor neurons, resulting in progressive skeletal muscle denervation, atrophy, particularly of the proximal muscles, and paralysis [3]. Moreover, we have recently demonstrated a selective reduction of layer V pyramidal neurons in the SMA murine model [4].

The disease is caused by homozygous deletions or mutations in the survival motor neuron gene, *SMN1*. In fact, the *SMN* gene is present in the human genome in two forms, *SMN1*, the ancestral telomeric copy, and *SMN2*, a centromeric paralogue that arose in recent evolution through duplication [5]. SMA patients lack *SMN1*, but always carry at least one copy of *SMN2*—a complete loss of *SMN* is incompatible with life. However, SMN2 protein is only partially functional: a critical, translationally silent single nucleotide C to T transition 6bp inside *SMN2* exon7 profoundly influences splicing, therefore, 90% of the protein is unstable and rapidly degraded [6,7] and is unable to compensate for the lack of SMN1 [8,9]. The severity of the disease is inversely proportional to *SMN2* copy number [10].

*SMN1* encodes a 38-kDa protein, that is expressed in all tissues and localized to the nucleus and cytoplasm [11] and has its primary and most prominent role in spliceosomal assembly and pre-mRNAs maturation. How reduced SMN levels lead to selective degeneration of motor neurons in SMA still remains elusive. The key role played by SMN in RNA processing suggests that splicing defects lead to breakdown of the neuromuscular system [12]. However, splicing defects occur only during late stages of the disease, and are unlikely to contribute to the pathogenesis [13]. Alternative hypotheses focused on the role of SMN in transport of mRNA in neurons [11], and on additional neuronal or muscle specific functions [14,15]. Furthermore, motor neuron subtypes differ strikingly from each other in the extent to which they are affected by SMA. Usually, the weakness is symmetrical and more proximal than distal. By contrast, several subsets are still intact at the disease endpoint [16].

Recently, micro RNA (miRNA) were shown to be a component of a novel class of ribonucleoprotein (RNP) complexes termed miRNPs [17], that contain the proteins Gemin3 [18] (a putative RNA helicase), Gemin4 [19] (a cofactor of Gemin3) and eIF2C2 [17] (a member of the argonaute family of proteins). Gemin3 and Gemin4 are also components of the SMN complex, a large multiprotein complex containing SMN, Gemin2 [20], Gemin5 [21,22], Gemin6 [23,24] and Gemin7 [25], that functions in the assembly/restructuring of RNPs and transcriptosomes [26]. Therefore, it is possible that specific or general changes in the activity of the miRNPs, due to possible redistribution or changes in the levels of Gemin3 and Gemin4, play a role in the development of SMA [27]. In particular, mice that do not process miRNA in spinal muscular neurons exhibit hallmarks of SMA and the skeletal muscle-specific (myomiR) miRNA-206, highly conserved in *Homo Sapiens*, *Rattus norvegicus* and *Mus musculus*, was found in the RNPs from mouse neuronal cells [27,28]. Furthermore, Williams and coll. [29] showed that miRNA-206 is dramatically induced in a mouse model of amyotrophic lateral sclerosis (ALS), a neurodegenerative disease with different etiology, characterized, similarly to SMA, by loss of motor neurons, denervation, atrophy and paralysis of target muscles [30,31]. In particular, they show that knocking-out miRNA-206 in the ALS mouse accelerates disease progression, and that miRNA-206 is required for efficient regeneration of neuromuscular synapses after acute nerve injury, at least, in part through histone deacetylase 4 (HDAC4) signaling pathways [29]. Interestingly, it has been recently shown that HDAC4 expression levels negatively correlate with the extent of muscle re-innervation and functional outcome in patients with ALS [32].

Therefore, seen the tight relationship between SMN and miRNPs complexes, and the neuroprotective role of miRNA-206 in the regeneration of the neuromuscular junction (NMJ) in ALS, we investigated the miRNA-206 pathway in a mouse model of intermediate SMA (SMAII), the SMNΔ7 mice, morphologically and molecularly analyzing the fibers of the quadriceps, a muscle affected in the early stage disease [33,34]. This is one of the most common SMA models: mice have a lifespan of about 2 weeks and impairment of motor behavior clearly detectable 5–6 days after birth (P5-P6). Different aspects of this model have been analyzed in a number of studies, and some discrepancies still exist on the number and subgroups of spinal motor neurons affected during disease progression [35]. Our results showed, in quadriceps of SMA mice sacrificed at P10, a decrease in the percentage of type II fibers (fast-twitch fatigable and fast-twitch fatigue resistant); an increase in atrophic muscle fibers and the accumulation of neurofilament (NF) in the pre-synaptic terminal of the NMJs. Furthermore, molecular investigation suggest a direct link between miRNA-206-HDAC4-FGFBP1, and, in particular, a strong modulation of this pathway, probably activated as survival endogenous mechanism, although not sufficient to rescue the integrity of motor neurons. We speculate that modulating miRNA-206 expression might regulate the neurodegenerative pathway, crucial to motor neuron function and survival. Therefore, miRNA therapeutics, based on extensive research into specific target interactions, holds much promise as future therapies for this neurodegenerative disease.

## Materials and Methods

### Animal Care and Use

All experimental procedures on live animals were performed in strict accordance to the European Communities Council Directive 86/609/EEC (November 24, 1986) Italian Ministry of Health and University of Turin institutional guidelines on animal welfare (law 116/92 on Care and Protection of living animals undergoing experimental or other scientific procedures; authorization number 17/2010-B, June 30, 2010): additionally an ad hoc Ethical Committee of the University of Turin specifically approved this study. All efforts were made to minimize the number of animals used and their suffering.

The original breeding pairs of SMNΔ7 mice were purchased from Jackson Laboratory (stock number 005025; Jackson Laboratories). They were originally generated in the laboratory of A. Burghes [36] and are homozygous for both transgenes (h*smn*2 and *smnΔ7*) that allow mice that are homozygous null for mouse *smn*, and would otherwise die embryonically, to survive to the end of the second postnatal week. To maintain the colony required the identification of mice heterozygous for deletion of *smn* (*smn* ^*+/-*^, carrier), that are fully viable, and their interbreeding. Mice afflicted with SMA are the homozygous mutant for *smn*: *smn* ^-/-^. The offspring were genotyped by PCR assays. Mice had free access to food and water. Data were obtained from knock-out SMA (*smn* ^*-/-*^, SMA) and wild type (*smn* ^*+/+*^, WT) mice sacrificed at P5 and P10, P11 and P12 (P10/12), considering P0 as the day of birth.

### Genotyping mice

DNA from mouse tail was extracted incubating a small piece of tail in 100μl of lysis buffer (10mM Tris HCl, 50mM KCl, 0.01% gelatin, 0.45% IGEPAL, 0.4% Tween-20) and 25μg of proteinase k at 55°C over night under gentle shaking [37]. The absence of the survival motor neuron gene *(smn)* was determined by PCR analysis using primers that amplify a portion of the *smn* gene, yielding a 420 bp product for the wild type (WT) allele and a 150 bp product for the knocked-out one (SMA). They were: smn fwd 5’-TTTTCTCCCTCTTCAGAGTGAT-3’, smn wt rev 5’-CTGTTTCAAGGGAGTTGTGGC-3’ and smn tg rev 5’-GGTAACGCCAGGGTTTTCC-3’ as suggested by supplier’s (Jackson Laboratories).

### Quadriceps histological examination

P10 mice (WT n = 17; SMA n = 10) were deeply anesthetized by gaseous anesthesia (3% isoflurane vaporized in O_2_/N_2_O 50:50) and perfused transcardially with 4% buffered paraformaldehyde (PFA, pH 7.4). Quadriceps were collected and postfixed in 4% PFA for 2 hours at 4°C. Samples were then transferred overnight into 30% sucrose in phosphate buffer (PB) 0.1M at 4°C for cryoprotection, embedded in cryostat medium (Killik) and cut on the cryostat (HM 550; Microm). Then, muscles underwent different histological procedures.

#### Hematoxylin/eosin staining

In order to morphologically evaluate the muscle (in terms of mean fiber area, quadriceps cross-sectional area, mean muscular fiber number), the quadriceps (WT n = 6; SMA n = 5) were cut on the cryostat in transverse 20μm thick sections, mounted directly onto 5% gelatin-coated slides, stained with hematoxylin/eosin, dehydrated in ascending series of ethanol (95–100%) and cleared in xylene. The sections were drawn and analysed by Neurolucida software (MicroBrightField Inc., VT) and data were obtained by the associated data analysis software NeuroExplorer (MicroBrightField).

#### Cytochrome c Oxidase (COX) assay

To assess fiber type composition, the samples (WT n = 5; SMA n = 3) were cut on the cryostat in transverse 50μm-thick sections free-floating in phosphate-buffered saline (PBS) and directly processed for the COX histochemistry. The reaction solution contained 10ml PBS 0.1M, 0.4g sucrose, 10mg DAB and 5mg cytochrome C (Sigma Aldrich). The solution was heated at 37°C before incubating the sections. Quadriceps were incubated with the reaction solution for 2–3 hours at 37°C on a tilting plate. We classified the fibers in the COX-stained muscle sections as follows: the dark ones were identified as oxidative fibers (type I), whereas light ones as glycolytic fibers (type II) [38].

#### Immunofluorescence

The samples (WT n = 5; SMA n = 7) were cut in longitudinal 20μm-thick sections mounted directly onto 5% gelatin-coated slides, immunoreacted and used to assess the NMJ innervation/denervation. Briefly, the tissue was permeabilized for 20 min in PBS-Triton 0.3% at RT on a tilting plate and then incubated with Alexafluor 555-conjugated α-Bungarotoxin (BTX) (1:500; Invitrogen) at RT for 30 min. After blocking non-specific binding sites for 30 min at RT with 0.3% Triton X-100 and 10% normal donkey serum (Sigma-Aldrich) in PBS 1X (pH 7.4), the sections were incubated with the monoclonal antibody anti-neurofilament 165 kDa (2H3 clone; mouse; 1:500; Hybridoma bank) in the same solution at 4°C overnight. After being washed in PBS 1X, the sections were incubated with secondary antibody Alexa488 (anti-mouse 1:200; Jackson Immunoresearch) followed by 4’,6 Diamino-2 phenyindole Dilactate (DAPI; Sigma Aldrich) in PBS 1:50 for 3 min. Samples were washed and coverslips were mounted with a drop of PB 0.1M with glycerol. The slices were analysed with a Nikon Eclipse 90i epifluorescence microscope and photographed by a Nikon DS-5Mc digital camera. In order to analyze the innervation, we evaluated the number of NF-positive nerves reaching each endplate at high magnification (100x objective): for each sample, about 100 NMJs were randomly evaluated [39]. We classified them as denervated, mono-innervated or multi-innervated. Moreover, for checking double staining and making 3D reconstructions, some preparations were examined also with a Leica TCS SP5 confocal laser scanning microscope. Additionally, some images were also analyzed using the isosurface module of Imaris software (Bitplane). Finally, we studied the endplate area and the NMJ density in WT and SMA mice (WT n = 3; SMA n = 3): all the BTX-positive endplates present in 20μm-thick longitudinal quadriceps sections were analyzed and drawn by Neurolucida software (MicroBrightField Inc., VT). Mean NMJ areas were calculated for both groups, and size distribution histograms (expressed in percentage) were constructed by grouping cross-sectional areas in 10μm^2^ bins. NMJ density has been expressed as a percentage of the endplate area covered on the total quadriceps area.

### Transfection

The myoblast cell line C2C12 (ATCC) was grown in RPMI supplemented with 10% FBS, penicillin (100units/ml) and streptomycin (100μg/ml) (Life Technologies). C2C12 cells were seeded at a density of 4x10^4^ (30% confluence) and 8x10^4^ (60% confluence) on 12-mm diameter plastic dishes and maintained at 37°C in a humified 5% CO_2_/95% air atmosphere. Twenty-four hours after seeding, they were transfected with 40pmols of miRNA-206/non targeting control miRNA (NT-miRNA, Bioneer) with lipofectamine 2000 following supplier’s instructions (Life Technologies). After a 4 hours incubation period, the medium was replaced with a fresh one. Forty-eight hours after transfection cells were collected for RT-PCR and WB analysis.

### RT-PCR analysis

C2C12 cells transfected with miRNA-206/NT-miRNA were collected in Trizol and total RNA was extracted following supplier’s instructions (Life Technologies). Quadriceps were quickly removed from P5 and P10, P11 and P12 mice after decapitation, then immediately frozen on dry ice and stored at -80°C until use (WT n = 14; SMA n = 15). Mice tissues were homogenated in liquid nitrogen and total RNA, enriched in miRNAs, was extracted with MirVana extraction Kit following supplier’s instructions (Life Technologies). The first-strand cDNA was synthesized with 0.5μg or 2μg of total RNA using the High Capacity cDNA Reverse Transcription Kit following supplier’s instruction (Life Technologies). Quantitative real-time PCR was performed with SYBR green core reagent kit for pri-miRNA-206 or TaqMan assays for mature miRNA-206 and FGFBP1 in a StepOne real time PCR system (Life Technologies). The primers used for pri-miRNA-206 were 5’-GTGTGTGGTTTTGGCAAGTG-3’ and 5’-GGGAGCATAGTTGACCTGAAA-3’ [29]; and for the Rpl26, used as housekeeping gene for data normalization, were 5’-CGAGTCCAGCGAGAGAAGG-3’ and 5’-GCAGTCTTTAATGAAAGCCGTG-3’ [40]. Samples were amplified simultaneously in triplicate in 1 assay run. Changes in mRNA levels were determined as the difference in threshold cycle (ΔCt) between the target gene and the reference gene.

We considered as a single group P10, P11 and P12 mice (P10/12), since we did not observed differences in terms of molecular expression of the gene analyzed. On the contrary, for P5 group of animals we used only muscles from pups sacrificed exactly five days after birth, seen that the change in miRNA-206 expression pathway observed arose in the time lapse between five and ten days after birth.

### Western blot

Total extract proteins were prepared from transfected C2C12 cells and from quadriceps homogenated in liquid nitrogen (WT n = 8; SMA n = 8). They were resuspended in ice-cold lysis buffer (20mM Hepes pH 7.5, 1%Triton, 150mM NaCl, 10mM NaF, 1mM Na_3_VO_4_, 0.5μg/ml apronitin, 1μg/ml leupeptin, 1μg/ml pepstatin) and then cleared by centrifugation. The supernatant was used for Western blot analysis after protein determination by the Bio-Rad protein assay (Biorad). Samples with equal protein concentration were resolved by SDS-PAGE on 4–12% NUPAGE gels using MES running buffer (Life Technologies) and subsequently blotted to nitrocellulose membrane (Amersham) To detect the proteins of interest, specific antibodies: anti-HDAC4 (rabbit polyclonal antibody, 1:500, Santa Cruz Biotechnology) and anti-GAPDH (mouse monoclonal, 1:1000, Millipore) were used. Immunoreaction was revealed using anti-mouse and anti-rabbit IgG conjugated to peroxidase, 1:2000 (Ge Healthcare) by the ECL reagent (Ge Healthcare). The optical density of the bands was determined by Chemi Doc Imaging System (Biorad) and normalized to the optical density of GAPDH.

### Statistical analysis

The data were evaluated as means ± standard error of mean (SEM). All data were expressed as absolute value, except the fiber type composition following COX histochemistry, the classification of NMJ innervation and the NMJ density, that were expressed as a percentage. For *in vivo* gene expression analysis, statistically significant differences among means were determined by ANOVA followed by post hoc Newman-Keuls test. For two experimental groups analysis (SMA vs WT), statistically significant differences among means were determined by t-test. The threshold for statistical significance data was set at p<0.05. Statistical analysis was performed using GraphPad Prism 6.0 software (GraphPad Software).

## Results

### Morphological analysis of quadriceps

**Fig 1:**
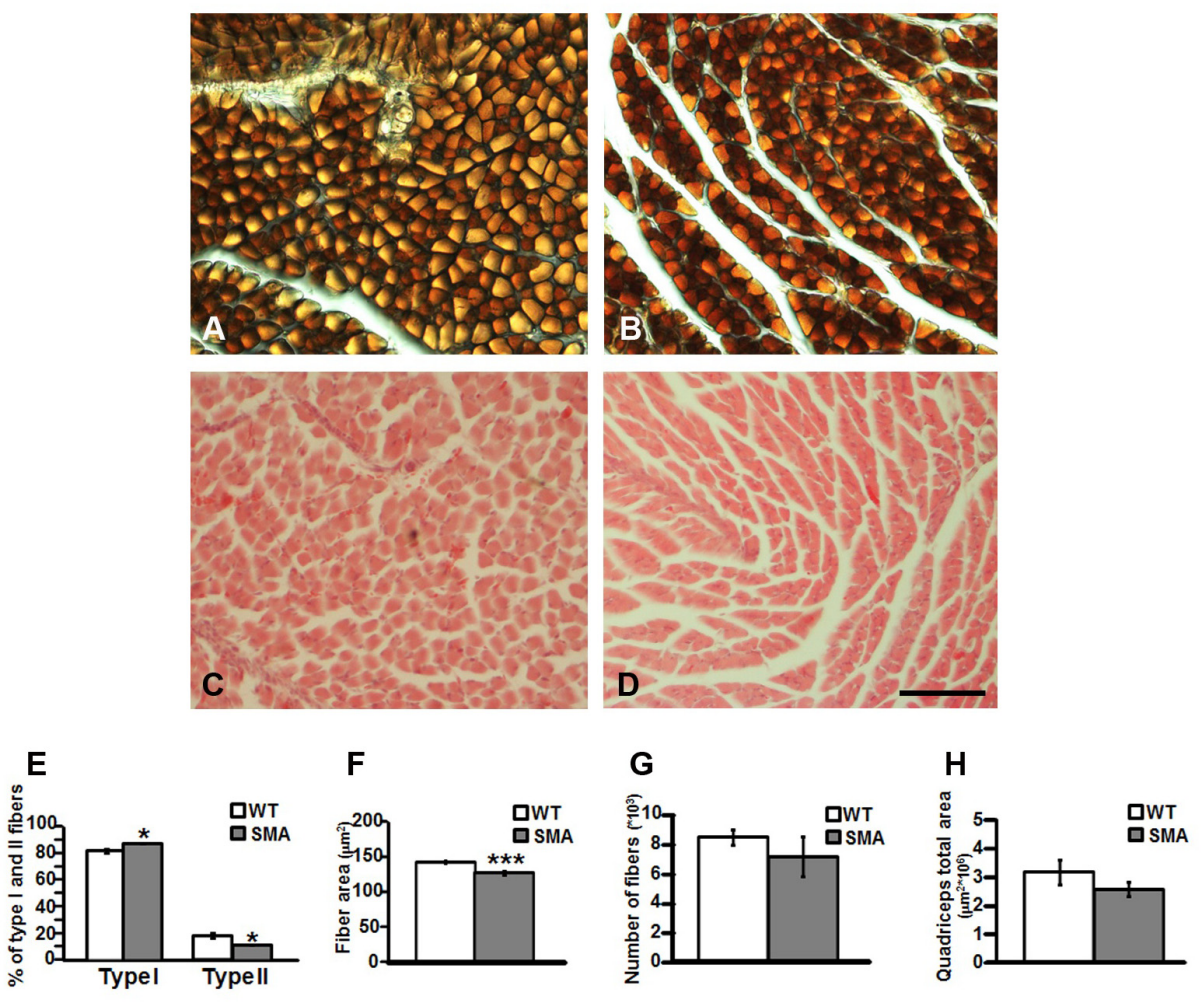
Fiber morphology of the quadriceps in WT and SMA mice at P10. Panels A-B, Cox immunoreaction on quadriceps of WT (panel A) and SMA (panel B) mice, revealing the mitochondrial activity and the fiber types. Scale bar = 40μm. Panels C-D showed the same muscles (WT: panel C; SMA: panel D) stained by hematoxylin/eosin. Scale bar = 80μm. Panel E, quantification of fiber type I and II in quadriceps of WT and SMA mice, expressed as percentage of the total number of fibers. Panels F-H, respectively single fiber area, number of fibers and total area of quadriceps in WT and SMA mice. *p<0.05; ***p<0.001 by t-test analysis.

**Fig 2:**
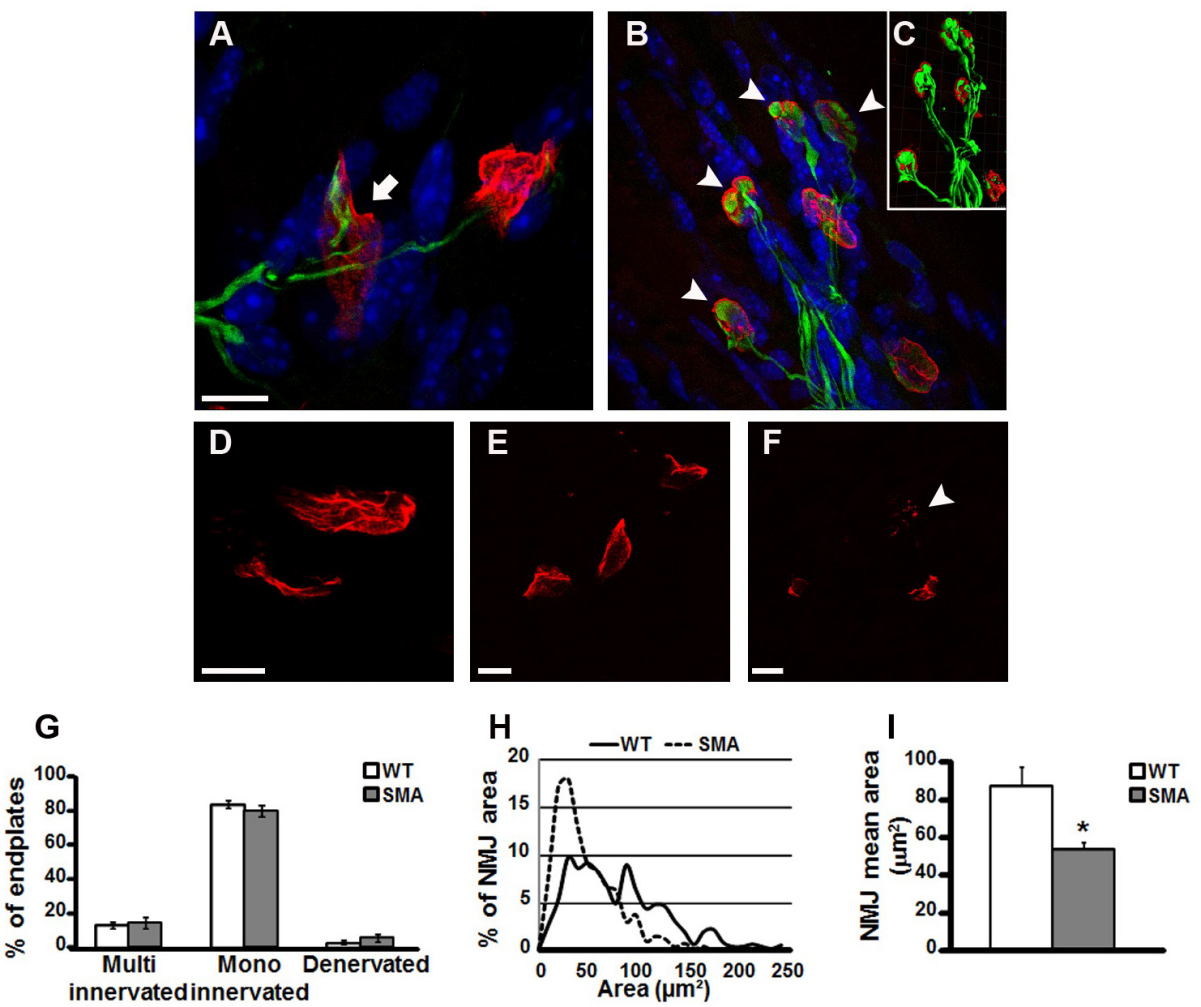
Architecture of the NMJ of the quadriceps in WT and SMA mice at P10. Panels A-B, Immunoreactions for α-bungarotoxin (red) and neurofilament (green) proteins in NMJs of quadriceps of WT (panel A) and SMA (panel B) mice. Inset C, isosurface module of Imaris software. Nuclei were labeled in blue with DAPI. The arrow in A points a WT NMJ correctly innervated, whereas arrowheads in B show the abnormal accumulations of neurofilament in SMA. Scale bar = 10μm. Panels D-F, morphology of NMJs at higher magnification: WT mice display endplates with a more mature morphology (panel D), compared to SMA ones (panel E). Additionally rare SMA NMJs appear fragmented, as indicated by arrowhead (panel F). Scale bar = 10μm. Panel G, quantification of multi-innervated, mono-innervated and denervated endplates in quadriceps of WT and SMA mice. Panel H, distribution of NMJ area in WT (continuous line) and SMA (dashed line) endplates expressed as percentage of total number of plaques. Panel I, mean value of NMJ area in WT and SMA quadriceps. *p<0.05 by t-test analysis.

We analyzed the morphology of quadriceps muscles, in WT and SMA mice at P10 by Cytorome c Oxidase (COX) histochemistry, hematoxylin/eosin staining (Fig 1), and the innervations of the NMJs by α-bungarotoxin and NF immunoreactions (Fig 2).

The COX reaction highlighted the mitochondrial activity of the different types of muscle fibers, therefore, we could classify them (Fig 1A and 1B). Our results showed a significant change in the fiber type distribution: type I fibers in WT mice were 82.00 ± 1.82% and in SMA mice 88.33 ± 0.33%). Consequently type II fibers were 18.00 ± 1.82% in WT and 11.67 ± 0.33% in SMA mice (Fig 1E).

After hematoxylin/eosin staining (Fig 1C and 1D), we analyzed the morphology of the muscle samples by Neurolucida software. We evaluated the mean fiber area, the mean number of muscle fibers and the overall quadriceps cross-sectional area in WT and SMA mice. We observed a statistically significant difference in the mean fiber area: WT 146.40 ± 2.35 μm^2^ vs SMA 127.39 ± 2.09 μm^2^ (Fig 1F). The other parameters showed a reduction trend in SMA compared to WT mice, but they did not show statistically significant differences. In particular, the mean number of muscle fibers was 8521.12 ± 514.59 in WT vs 7206.65 ± 1361.71 in SMA mice (Fig 1G), whereas the quadriceps total area was 3.21 ± 0.43 x10^6^ μm^2^ in WT vs 2.59 ± 0.26 x10^6^ μm^2^ in SMA mice (Fig 1H). Finally we checked the nuclei position into the SMA quadriceps and we did not observed centrally-nucleated fibers.

Furthermore, the analysis of the NMJ revealed dramatic abnormalities in the NMJs of SMA (Fig 2). Indeed, we observed altered NMJs, characterized by an aberrant NF accumulation (Fig 2A and 2C). We also observed that SMA NMJ postsynaptic terminals were simplified, with a decreased number of perforations compared to WT (Fig 2D and 2E). Rare endplates in SMA quadriceps also presented fragmentation (Fig 2F). Additionally, we classified the fibers according to their innervation: WT mice showed 2.75 ± 1.25% denervated fibers, 84.25 ± 2.39% mono-innervated fibers and 13.25 ± 2.14% multi-innervated fibers, compared to SMA mice 5.71 ± 2.22%, 80.29 ± 3.35% and 14.14 ± 3.38%, respectively (Fig 2G).

Finally, by Neurolucida software analysis, we compared NMJ area of WT and SMA mice. The analysis of the distribution of NMJ areas (bins = 10 μm) showed a dramatic shift to lower endplate size in SMA compared to WT mice (Fig 2H). In fact, the mean NMJ area was significantly smaller in WT mice compared to SMA quadriceps (87.21 ± 10.79 μm^2^ vs 53.58 ± 4.31 μm^2^, respectively; Fig 2I), while the NMJ density, expressed as a percentage of the endplate area covered on the total quadriceps area, did not differ significantly (0.182 ± 0.025% in WT vs 0.122 ± 0.007 in SMA mice).

### Molecular investigation of miRNA-206 pathway *in vitro* and *in vivo*

**Fig 3:**
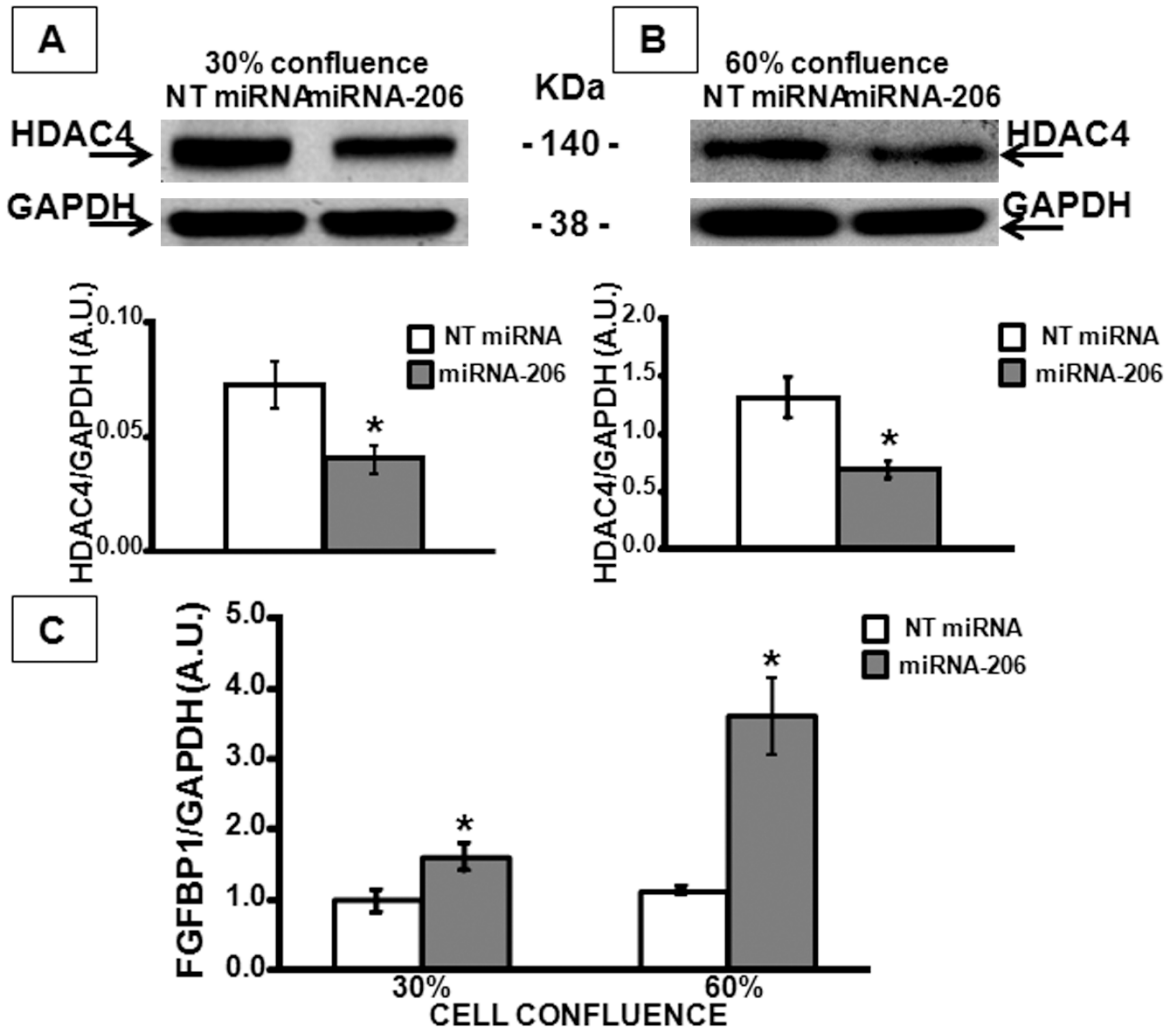
HDAC4 and FGFBP1 expression levels in C2C12 cells transiently transfected with miRNA-206. Panels A-B, representative Western blots displaying HDAC4 protein levels in C2C12 cells transfected at 30% confluence (panel A) and at 60% confluence (panel B) with a mimic miRNA-206 (miRNA-206) and with a non targeting miRNA (NT miRNA) used as control. Graphs below each image report the quantification of HDAC4/GAPDH protein levels. Panel C, evaluation of FGFBP1 mRNA levels in C2C12 cells transfected at 30% and 60% confluence with miRNA-206 and NT miRNA. *p<0.05 vs respective control by t-test analysis.

For molecular investigation, first of all we confirmed the existence of a miRNA-206-HDAC4-FGFBP1 pathway in C2C12 cell lines. In particular, we transiently transfected a miRNA-206 mimic and a non targeting miRNA as negative control (NT miRNA), in C2C12 at two different confluences, 30% and 60%. Forty-eight hours after transfection, cells were harvested and HDAC4 protein and FGFBP1 mRNA levels were measured by WB and real time PCR, respectively. Our results showed, in both experimental conditions, a down-regulation of HDAC4 (Fig 3A and 3B), as expected for a miRNA target, and a parallel strong up-regulation of FGFBP1 mRNA (Fig 3C), as a consequence of the miRNA mediated down-regulation of HDAC4 inhibitory effect on FGFBP1. Therefore, we could assume a direct link between miRNA-206, HDAC4 and FGFBP1 and decided to investigate the modulation of this molecular pathway in SMA quadriceps.

**Fig 4:**
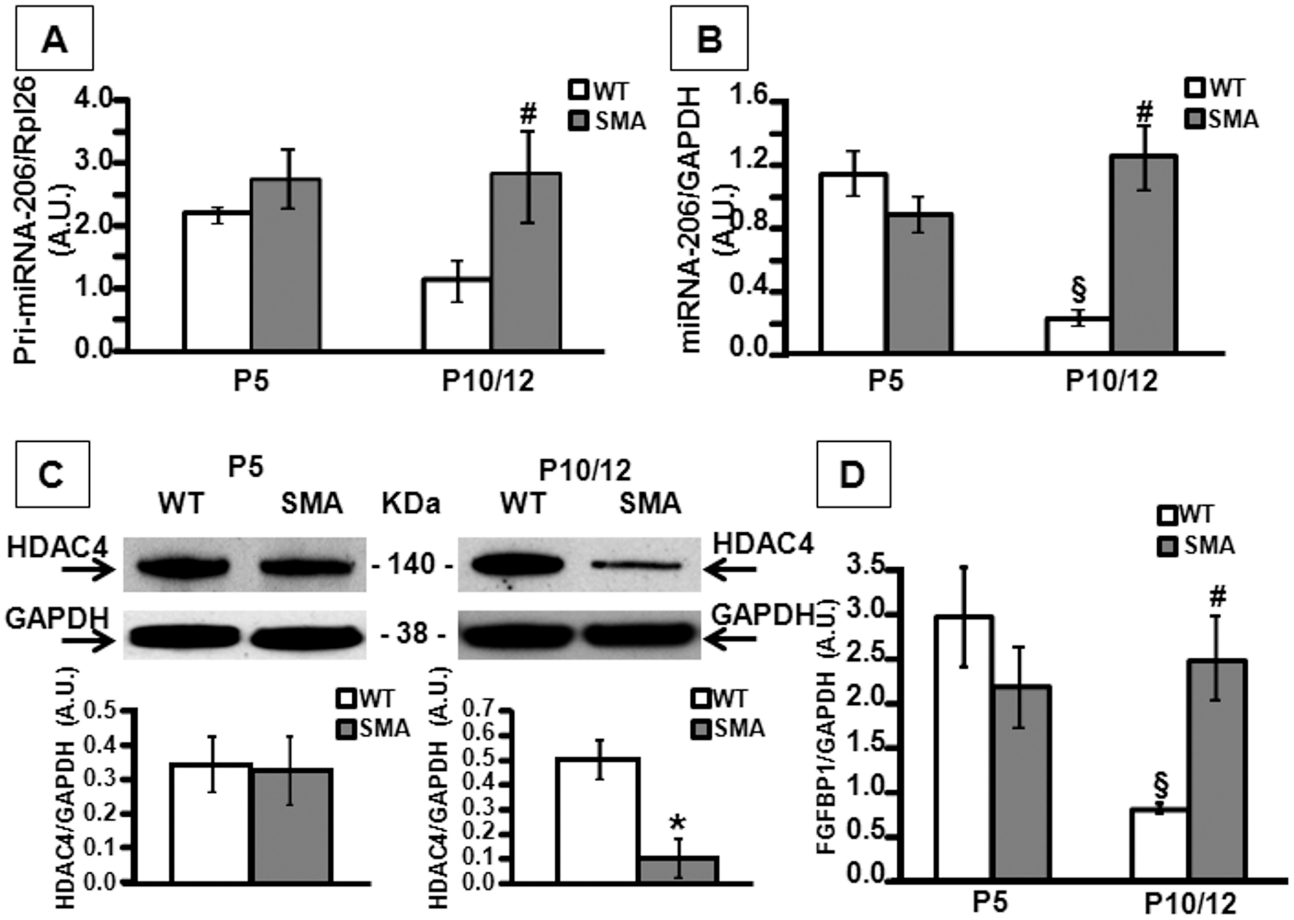
pri-miRNA-206, mature miRNA-206, HDAC4 and FGFBP1 expression levels in quadriceps of WT and SMA mice at P5 and P10/P12. Panels A,B,D, evaluation of pri-miRNA-206 (panel A), mature miRNA-206 (panel B) and FGFBP1 (panel D) mRNA levels in WT and SMA mice, five (P5) and ten (P10/12) days after birth. #p<0.05 vs P10/12 WT, § p<0.05 vs all group by ANOVA followed by post hoc Newman-Keuls test. Panels C, representative Western blots displaying HDAC4 protein levels in WT and SMA mice, at P5 (left blot) and P10 (right blot). Graphs below each image report the quantification of HDAC4/GAPDH protein levels. *p<0.05 vs WT by t-test analysis.

The molecular events leading to muscle fibers and NMJs alterations observed at P10 by morphological analyses might have taken place early in advance. Therefore, seen the overall differences in quadriceps of SMA mice at P10, we analyzed the expression levels of pri-miRNA-206 and mature miRNA-206 in a early stage of the disease (P5) and in an advanced stage of the disease (P10/12) by real time PCR in the quadriceps of both WT and SMA mice. Our results showed a strong up-regulation of both pri-miRNA-206 (2.5-fold increase) and miRNA-206 (5.6-fold increase) in SMA compared to WT mice only at P10/12 (Fig 4A and 4B). In particular, the physiological reduction in mature miRNA-206 occurring after birth in WT mice did not occur in SMA mice. Furthermore, the HDAC4 protein showed a significant down-regulation in the quadriceps of SMA mice at P10/12 and not a P5 (Fig 4C). Accordingly, the FGFBP1 mRNA was up-regulated in SMA mice (3-fold increase) only at P10/12, and the physiological decrease observed in WT mice from P5 to P10/12 did not occur in SMA, in agreement with miRNA-206 modulation (Fig 4D). On the other hand, in the gastrocnemius, we did not observe differences in miRNA-206 mRNA levels in SMA vs WT mice, excepted for the physiological down-regulation of miRNA-206 in SMA as well as in WT mice from P5 to P10/12 (data not shown).

## Discussion

### Quadriceps histological examination

In the present paper, we correlated morphological and molecular aspects of quadriceps, a muscle rich of slow-twich fibers (type I), in a mouse intermediate model of SMA, the SMNΔ7 mouse (SMA type II). Different aspects of this model have been analyzed but discrepancies regarding the NMJ architecture, the composition of the fibers, the extent of neuron loss and denervation still exist [39,41,42]. Our results, by COX histochemistry experiments, showed significant changes in the fiber type distribution in quadriceps sections of SMA mice sacrificed at P10. Although skeletal muscle fiber type distribution is quite heterogeneous, the high proportion of type I fibers in the quadriceps is probably due to the fact that these are postural and locomotive muscles that are active for a long duration, but at low contractile forces [43,44]. In particular, we found a 33% decrease in type II fibers (fast), and a parallel increase in type I fibers (slow) compared to age-matched WT mice, suggesting that slow motor units of the quadriceps are more resistant. These results are in agreement with studies of Dubowitz [45] conducted on muscle biopsies from severe SMA patients, showing widespread atrophy of type II (fast) fibers and a compensatory hypertrophic effect of type I (slow) fibers. Furthermore, several mouse model studies have concluded that fast fatigable motor units are intrinsically more vulnerable in ALS [46,47]. On the other hand, studies of Murray in SMAI (the most severe form of SMA) and SMAII mice revealed that motor neurons innervating the transverses abdominis (TVA), a predominantly slow twitch muscle, are more susceptible compared to the levator auris longus (LAL) and lumbrical muscles, exclusively fast-twich muscles [41]. In addition, motor neurons innervating the caudal band of the LAL are more susceptible than those innervating the rostral band, suggesting that motor neurons within a single homogeneous muscle, in terms of muscle fibers composition, can be differentially vulnerable. Therefore, Murray classified motor neurons according to their FaSyn/DeSyn characteristics, according to the initial focal clustering of postsynaptic AchRs, the timing of pre-synaptic maturation, and the maintenance of the NMJ in the adult mice [48], suggesting that FaSyn motor units are more vulnerable [41]. In addition, studies of Ling [39] on SMAII mice revealed that many but not all vulnerable muscle are predominantly fast-twitch, and that several FaSyn muscles are resistant to denervation, indicating that both classification, according to muscle fiber type or synapsing phenotype, cannot be the only determinants of NMJ vulnerability. In conclusion, differences in vulnerability based on motor neuron properties and muscle fiber type exist as our and others studies showed, although these parameters are not the only ones to be considered to predict motor neuron vulnerability.

Next, by hematoxylin/eosin staining, we observed a significant 15% reduction in the area of the single muscle fibers in agreement with previous results. In particular, Lee and coll. [49] reported in the same animal model a severe and uniform reduction in the size of sternomastoid, extensor digitorum longus and soleus muscles. According to that, muscle shrinkage was also observed in the transversus abdominis at P7, with a higher degree at P14, in the levator auris longus muscles at P14 [41] and in the tibialis anterior at P9, reaching higher levels at P13 [42]. Therefore, in an advanced phase of the disease, muscle shrinkage seems to be a widespread event in SMA fibers [50,51], responsible for the profound muscle weakness observed in SMA mice. In fact, the myofiber contractile strength is determined by its diameter [52] and by the composition of myosin heavy chains [53]. Finally, we checked the presence of centrally nucleated fibers, since this is considered a common pathological feature in a number of myopathies (as myotonic dystrophy and limb-girdle muscular dystrophies), but we did not detected myofibers with centralized nuclei. Indeed, such phenomenon is a very rare event in this SMA model, as confirmed by Avila’s group, that has conversely used this parameter as a sign of regeneration, to be checked after a treatment [54].

### Quadriceps immunohystochemical examination

Finally, by immunohystochemistry analysis we revealed a deep alteration of the NMJs, in terms of neurofilament accumulation and endplate area, but we did not observe a significant degree of denervation of the synaptic terminals. In agreement with our results, NF accumulation was also observed in the transversus abdominis and in the levator auris longus muscles of SMNΔ7 mice at P7 [41], in the flexor digitorum brevis 2 and 4 already at P1 [39], and in the extensor digitorum longus, the gastrocnemius, the tibialis anterior and the sternomastoid muscles at P15 in a different SMNΔ7 mouse model, where the mutation is restricted to neurons, that has a lifespan of 30 days [3]. Therefore, NF accumulation at the pre-synaptic terminal of the NMJ, a morphological hallmark of SMA, probably represents an early event in the pathogenesis. Nevertheless, NF accumulation did not seem to correlate with NMJ denervation. In fact, we did not observe significant differences in the number of denervated, mono-innervated, and multi-innervated plaques in SMA compared to WT mice at P10, in agreement with the minimal denervation observed in the quadriceps of SMA mice at P13 by Kong and coll. [42], and the recent studies of Ling and coll. [39]. Indeed, Ling found denervation in the majority of the NMJs of axial muscles investigated and in few appendicular muscles, but not in the quadriceps of end-stage SMA mice.

Furthermore, our data showed that NMJs of SMA quadriceps were significantly smaller and immature compared to WT, while the overall density of plaques did not significantly differ. Our results are in agreement with previous works demonstrating that SMA nerve terminals of the sternomaistoid at P15 and the tibialis anterior at P5, P9 and P13 are smaller and immature [42,49]. In fact, postsynaptic AChR aggregates initially appear as simple plaques and then become more complex as perforated plaques, and eventually mature with pretzel-like morphology [55]. Our results showed that in the quadriceps of SMA mice, this differentiation is incomplete, with less perforations and also fragmentation in some endplates. In particular, synaptic dysfunction, due to a decreased density of synaptic vesicles released at motor nerve terminals, results in reduced quantal content of neurotransmission [42,56,57]. These features are consistent with an abnormally persistent expression of embryonic type of AChRs (γ/fetal vs ε/adult subunit of AChR) at SMA NMJs that precedes axonal degeneration [42,49]. We did not detect significant differences in the density of NMJs, probably because to a reduced size of endplates area corresponds a smaller total area of quadriceps in SMA mice.

Overall, our studies confirmed and provided novel information on the deep morphological alterations of SMA quadriceps.

### Investigation of miRNA-206 pathway

We investigated the molecular pathway of miRNA-206 in the quadriceps, since its role emerged in ALS disease [29]. SMA and ALS have very different etiology, but are both characterized by progressive paralysis due to selective death of lower [58] and upper motoneurons [4]. Furthermore, among the miRNAs that are predominantly expressed in skeletal muscle, miRNA-206 has been defined as a key promoter of myogenic commitment [59,60]. Its expression is induced by MyoD, a critical transcriptional factor for muscle differentiation [61]. Augmenting the pro-myogenic effect of miRNA-206 has been considered as a therapeutic strategy to delay not only ALS [29], but also rhabdomyosarcoma [62] and muscular dystrophies [63,64,65]. Our results showed a miRNA-206 up-regulation in SMA quadriceps at P10/12, in an advanced stage of the pathology, while at P5, an early phase, we did not observe differences in miRNA-206 levels compared to WT muscles. Furthermore, in the gastrocnemius, a distal muscle affected later during pathology [50], we did not observe a miRNA-206 up-regulation, but on the contrary the physiological decrease observed after birth in WT mice. In fact, previous studies showed that, during embryonic development in the mouse, miRNA-206 is first detected at a very low level as early as E9.5 and thereafter begins to significantly increase [66]. Post-natal expression of miRNA-206 appears to peak at three days after birth, then it declines [60]. Down-regulation of HDAC4 protein levels have also been observed in SMA quadriceps at P10/12 compared to age matched WT mice. In fact, HDAC4 is a miRNA-206 target [29,67], as predicted by computational analysis [68,69]. Furthermore, Williams and coll. [29] show that FGFBP1 was down-regulated in miRNA-206 knock-out mice and up-regulated in HDAC4 knock-out mice, suggesting a strict correlation between miRNA206-HDAC4 and FGFBP1; our results confirmed a miRNA-206-HDAC4-FGFBP1 axis in the same *in vitro* model. In fact, over-expression of miRNA-206 in C2C12 cells by miRNA mimic transfection strategy, clearly showed a decrease in HDAC4 protein levels and resulted in FGFBP1 transcriptional up-regulation, as expected following down-regulation of HDAC4 inhibitory effect on FGFBP1 mRNA.

### Role of HDAC inhibitors and FGFs in SMA

HDAC4 is a class II histone deacetylase, highly expressed in striated muscle [70], implicated in the regulation of cardiac and skeletal muscle growth [71]. Interestingly, inhibitors of HDACs, such as suberoylanilide hydroxamic acid (SAHA) [72], trichostatin A (TSA) [54], phenylbutyrate [73], sodium butyrate [74], valproic acid [75], and hydroxyurea increase *SMN2* full length transcript and have been demonstrated to ameliorate SMA phenotype at least in mouse models [76,77]. HDAC4 has been well characterized for its ability to repress the myogenic transcription factor Mef-2 [78], induce *myogenin* expression through the repression of *Dach2* gene, a repressor of *myogenin* [79,80]. In particular, up-regulation of HDAC4 protein by down-regulation of miRNA-206 expression, promotes hypertrophy of cultured myotubes [81]. On the other hand, over-expression of miRNA-206 *in vitro* and *in vivo*, did not result in muscle atrophy. Hence, miRNA-206-HDAC4 axis did not alter skeletal muscle mass of young adult mice [81]. Therefore, we speculated, consistent with Williams’ hypothesis [29], that FGFBP1 is the key protein downstream HDAC4, responsible of miRNA-206 neuroprotective actions. In fact, FGFBP1 interacts with FGF-7, FGF-10 and FGF-22 family members and potentiates their bioactivity by releasing them into the extracellular matrix [82]. FGFs are muscle-derived regulators that promote presynaptic differentiation of the NMJ [83]. For this reason, we speculate that endogenous miRNA-206 up-regulation, with consequent HDAC4 protein reduction and FGFBP1 mRNA increase might be a neuroprotective mechanism activated by muscle cells to increase re-innervation of muscle endplates, however not sufficient, to rescue motor neuron from death. The late activation of this neuroprotective mechanism might be correlated to the late denervation of the NMJs. On the contrary, to treat animals in the very early stage disease would very important for therapeutic efficacy. Indeed, previous studies with self-complementary adeno-associated virus 9 to delivery *SMN* (scAAV9-SMN) in SMA pups, demonstrated that injection of scAAV9-SMN at P1 rescues motor function, neuromuscular physiology and increases lifespan of mice; at P5 results in partial correction; at P10 the treatment has little effect [84]. According to this study, TSA administration at P5, but not at P10, increases SMN and SMNΔ7 proteins [54].

### Future perspectives in SMA therapy

We speculate that increasing miRNA-206 levels in quadriceps muscles, in a pre-symptomatic phase of the pathology, can protect motor neurons, improve motor ability and life expectancy of transgenic mice, similar to the neuroprotective effect observed in ALS mice [29]. Therefore, further studies are necessary to unravel this important aspect. Indeed, future experiments will be drawn to up-regulate miRNA-206 levels in muscles of SMA mice to investigate its neuroprotective role and to characterize the complete molecular pathway downstream miRNA-206 activation. A list of putative miRNA-206 target genes compiled from TargetScan, PicTar and Miranda databases revealed an over-representation of genes associated with protein modification, transport, cytoskeleton organization, RNA metabolism and transcription [69]. However, further studies are required to investigate other molecular targets of miRNA-206 that could be relevant in motor neuron diseases. In fact, any given miRNA may have hundreds of targets, therefore altering the cellular level of a single miRNA has the potential to regulate multiple cellular pathways simultaneously. This property can be advantageous as a means to modulate a disease process in its entirety. In particular, miRNA therapeutics for neurodegenerative diseases represent a growing field of research as a therapeutic approach. The first miRNA-targeted drug that enters phase II clinical trials is miravirsen, that specifically binds to miRNA-122 and effectively decreased hepatitis C virus replication [85]. The mimic of miRNA-34a, a tumor suppressor miRNA, entered phase I clinical trials [86]. Many more miRNA therapeutics are expected to enter clinical trials as new disease targets are identified and new sequences show activity in diseases.

### Conclusions

Overall, our morphological data confirmed muscle shrinkage in SMA fibers and profound alterations of the NMJ, not correlated with denervation of the synaptic terminals; and provided novel information regarding the fiber type distribution in quadriceps of SMAII mice at P10. Furthermore, our molecular investigation confirmed a direct link between miRNA-206-HDAC4 and FGFBP1 *in vitro* and *in vivo*, and that this pathway is up-regulated in a late phase of the disease, suggesting that miRNA-206 could be an innovative, still relatively unexplored, strategy to treat SMA. Future studies will be aimed at specifically addressing these questions.
